# Bilobed spleen, transposition of the inferior vena cava and Riedel lobe: an extremely rare imaging finding in the same case

**DOI:** 10.1259/bjrcr.20180091

**Published:** 2018-12-05

**Authors:** Mohamed Salah Elfeshawy

**Affiliations:** 1 Department of Radiology, Al Azhar University, Faculty of Medicine, Al Hussein University Hospital, Cairo, Egypt

## Abstract

There is a wide range of congenital anomalies of the spleen regarding its shape, location, number, and size. Most of these congenital anomalies are commonly detected on ultrasonography, CT, or MRI and may sometimes represent a challenging diagnosis for radiologists and clinicians. The bilobed spleen is an extremely rare form of congenital anomaly. In most cases, it is accidentally discovered during abdominal surgeries. The bilobed spleen is usually large in size when compared with the normal spleen; hence, it is more liable to trauma. Transposition of the inferior vena cava (IVC; also known as left-sided IVC) refers to a very rare variant course of the IVC. The most common variations are duplicate IVC, as well as retroaortic left renal vein and circumaortic venous rings. Left-sided IVC occurs in 0.17–0.5% of the general population. Diagnosis of left-sided IVC is important when planning vascular procedures like portosystemic shunt, the placement of an IVC filter, nephrectomy, and renal transplant. There should be an awareness of the Riedel lobe, which is a common anatomical variant of the liver, as it can simulate a mass. Its misidentification as a pathological abdominal mass can lead to surgery; pathology can also occur (*e.g.* malignancy or even torsion). In this report, we presented a case of a bilobed spleen that was misdiagnosed as a left renal mass during routine abdominal ultrasonography in a 25-year-old female who complained of recurrent left hypochondrium pain. The bilobed configuration was confirmed with MRI and ultrasound examination of the abdomen.

## Background

The spleen is a large, encapsulated organ that mainly encompasses vascular and lymphoid tissue; it is situated in the upper-left quadrant of the abdominal cavity between the diaphragm and fundus of the stomach, and it relates to the tail of the pancreas and the upper pole of the left kidney. It is important to understand the wide variety of splenic shapes, numbers, fissures, and positions to avoid imaging pitfalls and to safeguard against misinterpreting these normal variations as pathological processes.

A persistent left inferior vena cava (IVC) is caused by the regression of a right supracardinal vein and the persistence of a left supracardinal vein. Typically, the left IVC meets the left renal vein, which passes anteriorly to the aorta in an “N fashion”, thereby joining the right renal vein to create a normal right-sided prerenal IVC;^1^ in the literature, the left IVC has a prevalence of 0.17–0.5%.^2^

Here, we report an extremely rare case of multiple congenital anomalies including a bilobed spleen, where there is medial (internal) and lateral (external) splenic lobes connected at the splenic hilum. In this case, we present a left IVC that was draining into the hemiazygos vein that eventually joined the azygos vein, supradiaphragmatically. The combined hemiazygos and azygos veins were draining into the superior vena cava (SVC). This pattern is not consistent with the common left IVC variations reported in the literature. In fact, in the literature, we were not able to find data on the prevalence of left hemiazygos/azygos variations.^2^ We believe that a left IVC with hemiazygos and azygos continuation is a very rare variation of IVC.

The size of the liver depends on several factors such as age, sex, body size, and shape. The liver can be palpable for anatomical reasons or due to underlying abnormal conditions,^3^ and congenital abnormalities of the liver are considered rare.^4^ The Riedel lobe of the liver is one such variation; it is a tongue-like inferior projection of the right lobe of the liver that extends to the right of the gall bladder.^5^

A Riedel lobe was reported in seven female patients who had palpable masses in the right hypochondrium, and the diagnosis was confirmed at surgery.^4^ Riedel lobes should be included in the differential diagnosis of right-sided abdominal palpable masses to avoid unnecessary surgery.^6^

## Discussion

Careful examination of the spleen should be done to differentiate between commonly encountered congenital anomalies and different pathological entities. The spleen is normally located in the upper-left quadrant of the body; it does not develop directly from the gut. Several suspensory ligaments, including gastrosplenic, lienophrenic, lienocolic, and lienorenal ligaments maintain the spleen in its normal position.^7^

The spleen undergoes a number of developmental processes that result in a wide range of anatomical differences, which can include variations in shape, as well as the presence of fissures and notches. The spleen originates from the mesenchyme of the dorsal mesogastrium, which lies over the dorsal pancreatic endoderm as a long strip of cells next to the developing stomach. The spleen loses its hematopoietic function on embryo development. In late pregnancy, lymphoid precursor cells migrate into the spleen from the central lymph organs. The splenic notch is a remnant of the previously lobulated structure of the spleen.^8^

The spleen is usually a single organ, but it is commonly surrounded by smaller amounts of splenic tissue (splenunculi or accessory spleens), which usually lie in proximity to the pancreatic tail.^9^

Splenunculi are more frequently detected in a number of advanced imaging modalities; thus, they can be differentiated from more sinister pathologies.^10^ It is easy to differentiate splenunculi from enlarged lymph nodes, as they display isodensity, as well as specific intensity and enhancement patterns on pre- and multiphasic post-contrast studies.^11^

The bilobed spleen may mimic enlarged lymph nodes and tumors in the left hypochondrium organs, which include hypervascular pancreatic tumors, neuroendocrine tumors, metastatic lesions, or enlarged lymph nodes in the splenic hilum.^12^ It should be differentiated from splenunculus.

Accessory spleens are small nodules of splenic tissue found distal from the main body of the spleen; this separation is the primary feature that differentiates it from the medial (internal) splenic lobe of the bilobed spleen, which is connected to the splenic hilum.^13^

The medial/internal lobe of the bilobed spleen can extend medially to displace and compress the pancreatic tail and the upper pole of the left kidney with a concomitant, non-existent lienorenal ligament. It can indent the posterior aspect of the gastric fundus, mimicking an iceberg tumor.^13^

Ultrasound, CT, and MRI remain the primary imaging modalities used to visualize the splenic parenchyma. MRI is preferred over CT scans given that there is no need for radiation exposure or contrast injection, and it offers better soft-tissue discrimination. The characteristics of the spleen visible via MRI are unique; there is a large amount of fractional heme content characterized by long *T*_1_ and *T*_2_ (*i.e.* it is lower in signal intensity than the liver on *T*_1_ weighted images and higher on *T*_2_ weighted images).

Ultrasound is a non-invasive, highly sensitive, and specific imaging technique that can be used to evaluate the spleen. Ultrasound evaluation of the spleen should not be limited to splenic size; rather, it should be used to determine its shape and location.^13^

**Figure 1.**
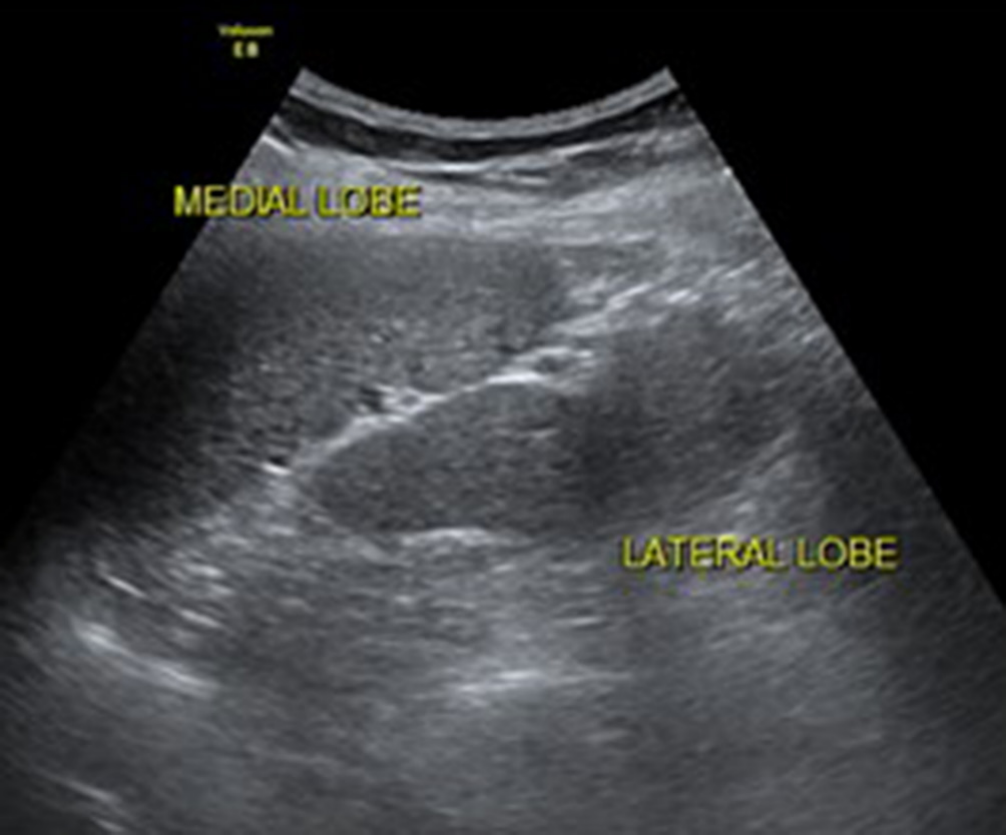
Long-axis ultrasonography image of the spleen showing a bilobed spleen with intervening splenic hilar vessels.

**Figure 2.**
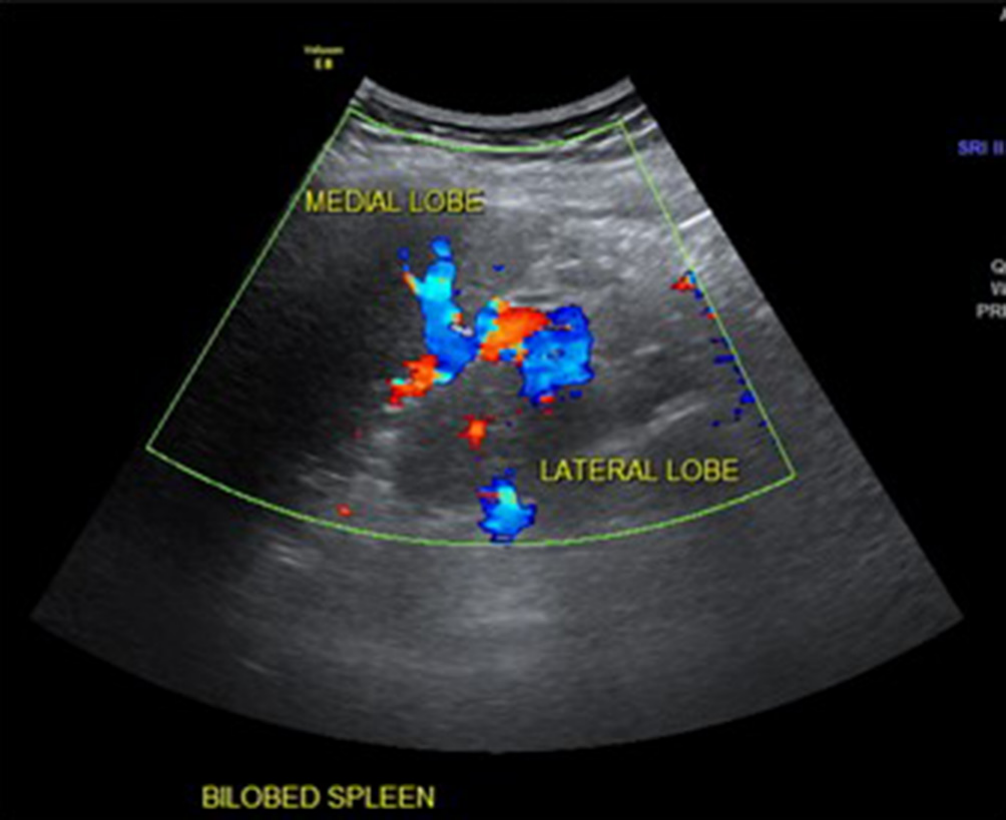
Long-axis color Doppler ultrasonography images of the spleen showing the intervening splenic hilar vessels between both lobes, as well as an enriched vascularity of both lobes.

**Figure 3.**
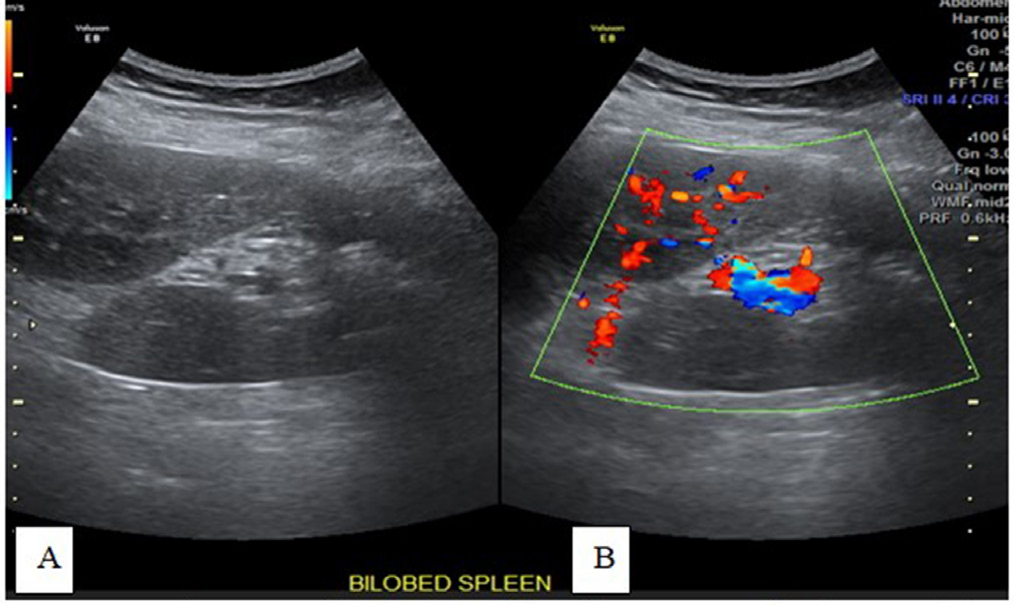
Transverse-axis gray and color Doppler ultrasonography images (A and B) of the bilobed spleen showing the intervening splenic hilar vessels between both lobes and enriched vascularity of both lobes.

The spleen is the dark male of the abdomen more echogenic than both the liver and kidney; it is best assessed in the supine, left lateral position with the left arm placed behind the head, obliquely in the 9th or 10th intercostal spaces (Figures 1–3).

In the current case, an ultrasound was performed, which revealed a bilobed spleen and small spenules (Figures 1–3); the left-sided IVC was draining into the hemiazygos vein that was eventually joining the azygos vein supradiaphragmatically. Moreover, the combined hemiazygos and azygos vein was draining into the SVC and enlarged caudate lobe, protruding into the left side.

The clinician did not concur that the mass was a medial splenic lobe and considered it a lesion arising from the left kidney; the clinician then compared the current ultrasound scan to a previous ultrasound that was performed by another radiologist who, unfortunately, misdiagnosed it as a left renal mass (and did not notice the other findings). The clinician asked for a CT scan with contrast, but the patient refused as she was waiting to get pregnant. The clinician then asked for an MRI scan without contrast and the patient consented.

**Figure 4.**
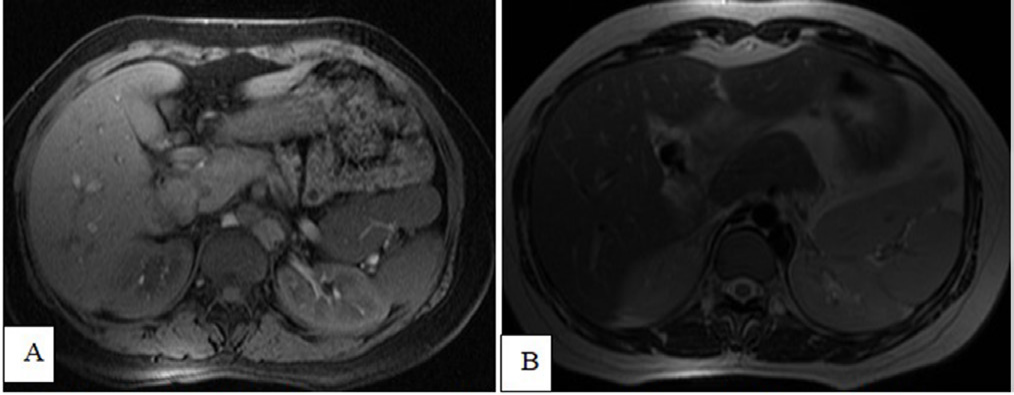
Axial T1 fat-saturation-weighted images (A) and *T*_2_ weighted imaging (B) showing a bilobed spleen indenting the upper pole of the left kidney.

**Figure 5.**
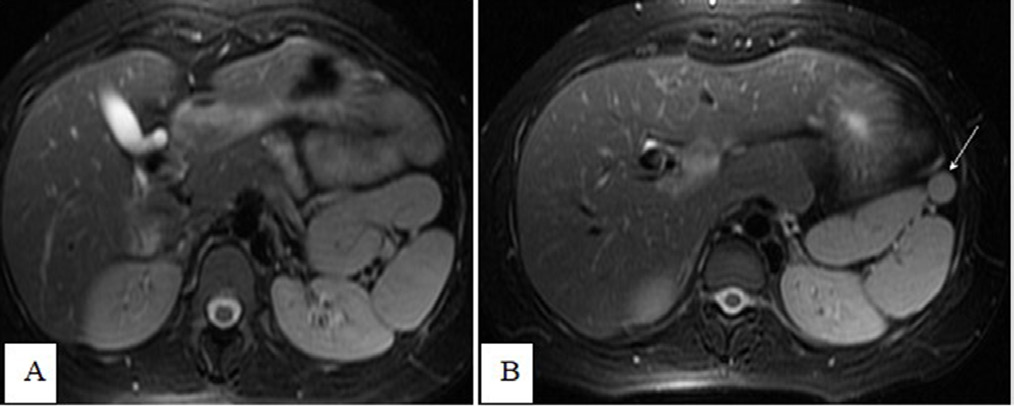
Axial T2 fat-saturation-weighted images (A and B) showing a bilobed spleen indenting the upper pole of the left kidney, as well as a small spenule (white arrow).

**Figure 6.**
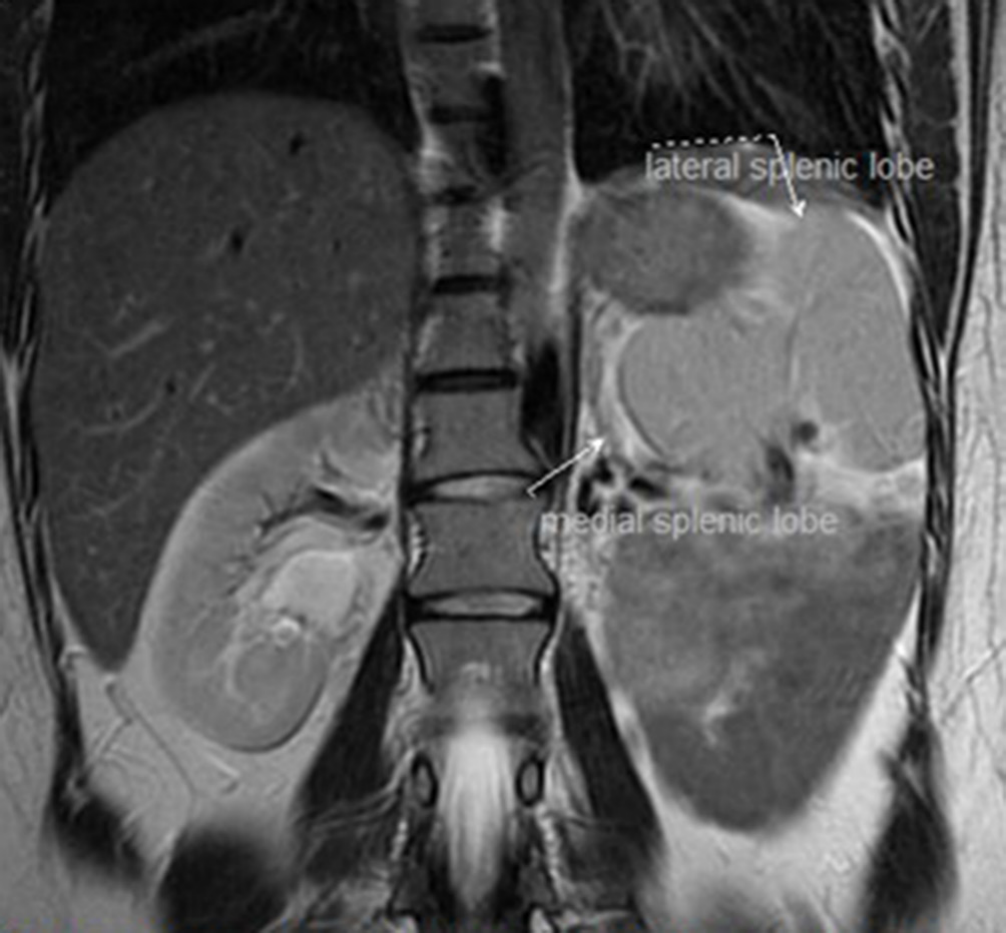
Coronal *T*_2_ weighted imaging showing a bilobed spleen.

On unenhanced MRI scans, the spleen is unique with a large amount of fractional heme content, characterized by long *T*_1_ and *T*_2_ weighted imaging (*i.e.* it is lower in signal intensity than the liver on *T*_1_ weighted images and higher on *T*_2_ weighted images) (Figures 4–6).

**Figure 7.**
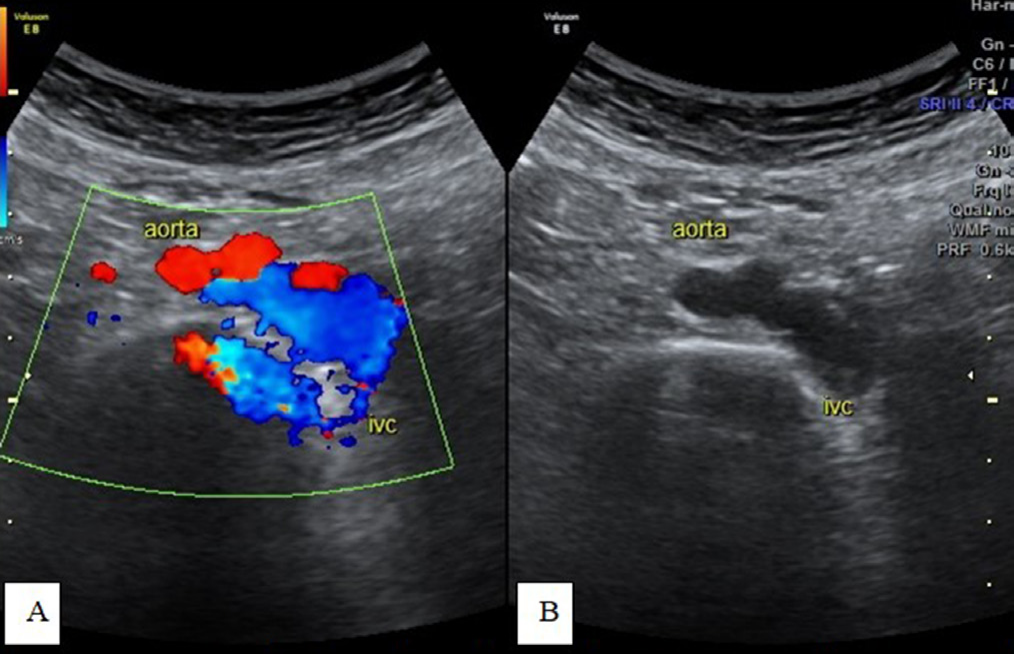
Transverse-axis color Doppler and greyscale ultrasonography images at the umbilical region showing a left-sided IVC. IVC, inferior vena cava.

**Figure 8.**
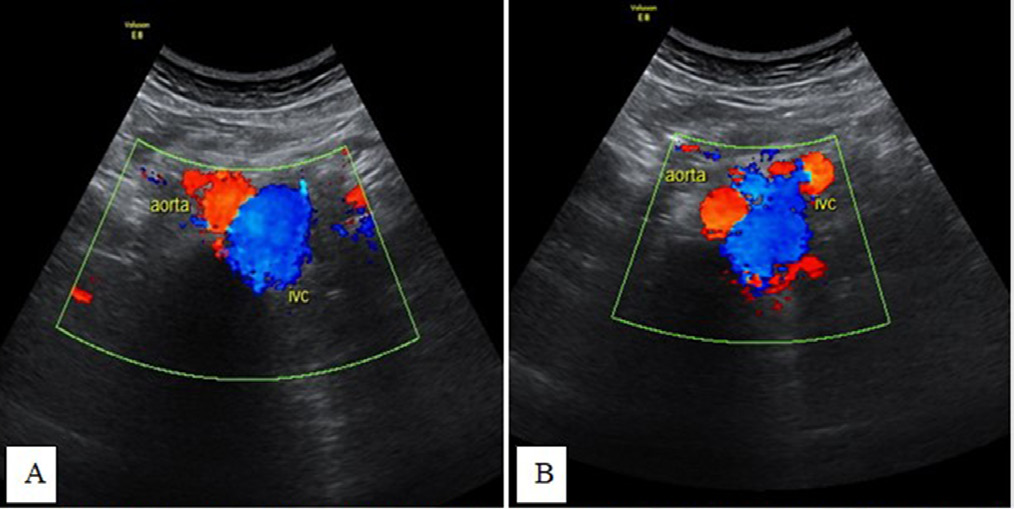
Transverse-axis color Doppler ultrasonography images (A and B) at the epigastric region showing a left-sided IVC with changes in size during respiration. IVC, inferior vena cava.

**Figure 9.**
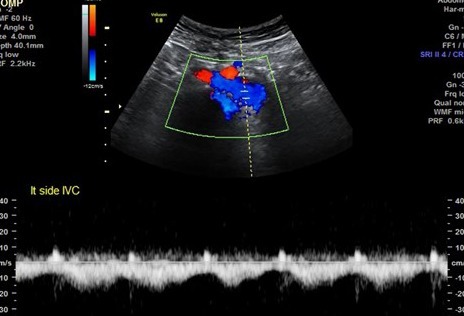
Pulsed Doppler ultrasonography image at the epigastric region showing a left-sided IVC with a venous waveform pattern. IVC, inferior vena cava.

**Figure 10.**
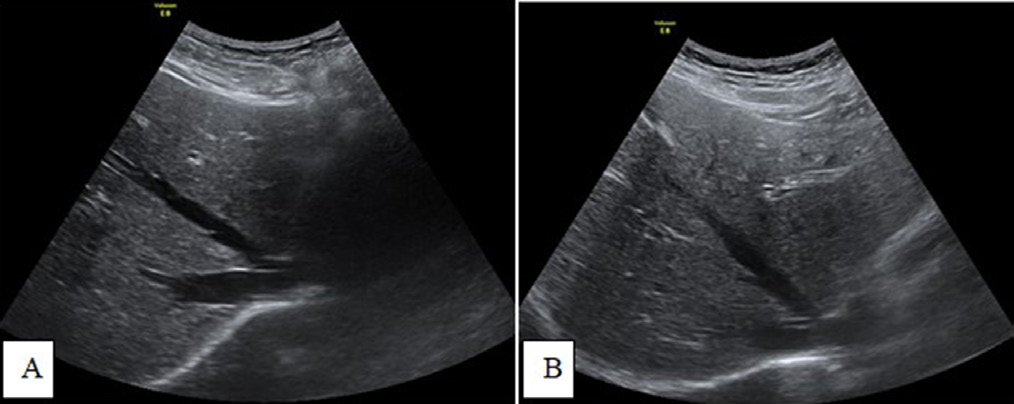
Transverse-axis ultrasonography images (A and B) showing an absent intrahepatic portion of the IVC and the hepatic veins that drain directly into the right atrium. IVC, inferior vena cava.

**Figure 11.**
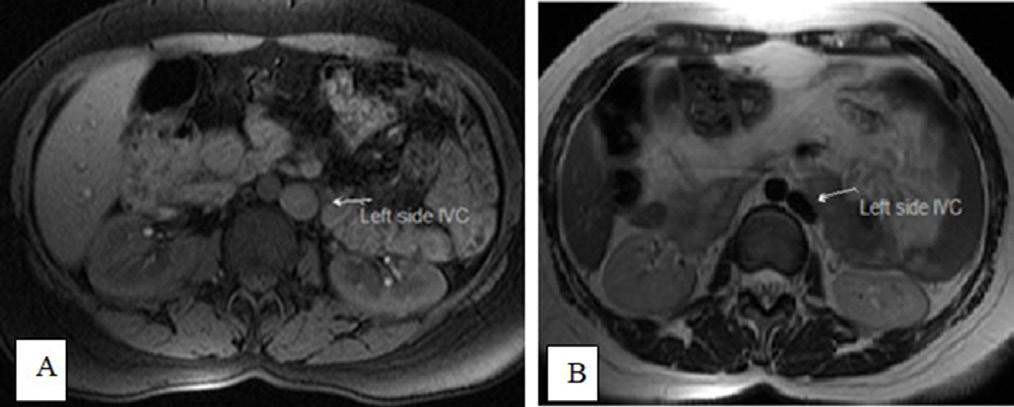
Axial *T*_1_ fat-saturated (A) and axial *T*_2_ (B) images showing a left-sided IVC. IVC, inferior vena cava.

**Figure 12.**
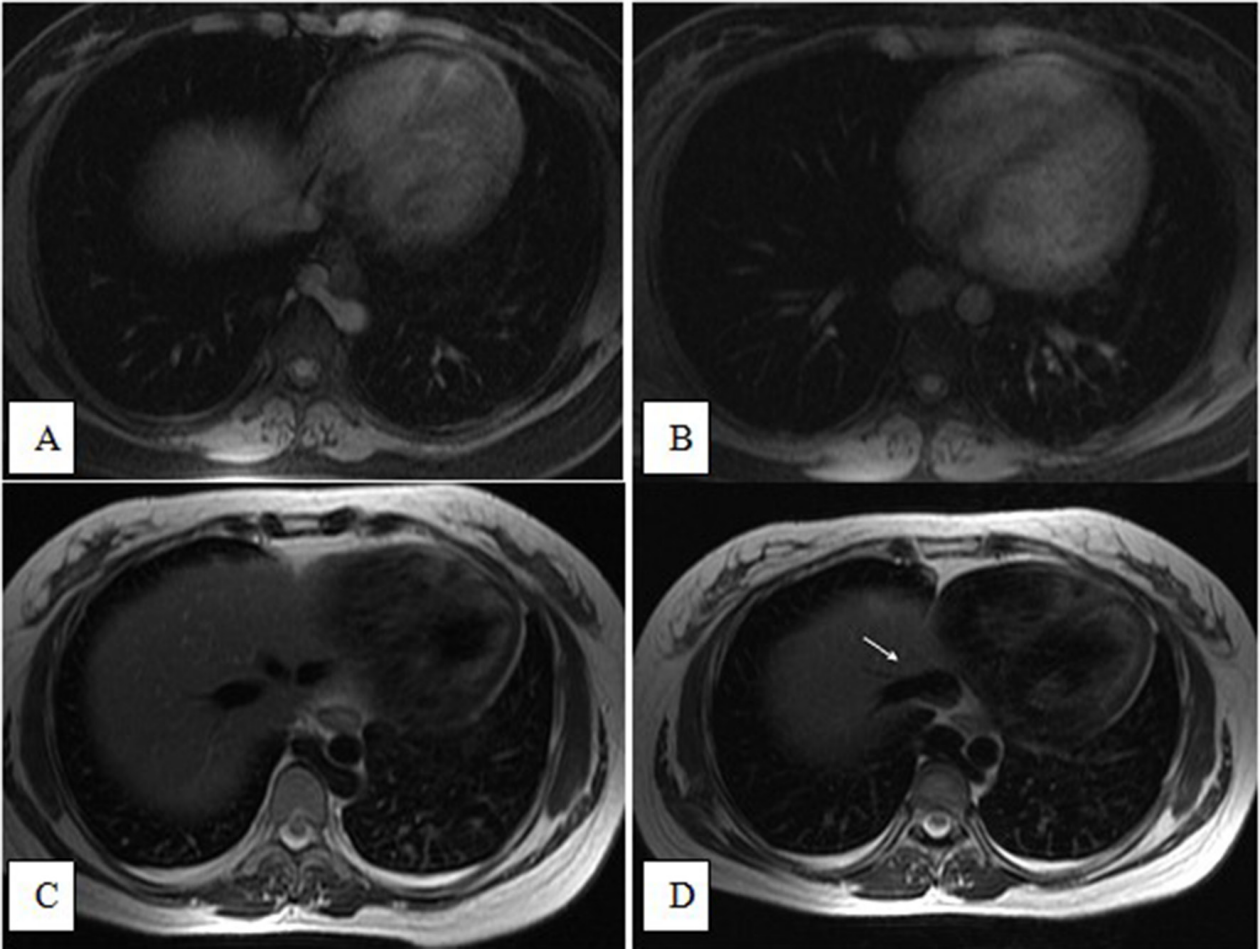
Axial *T*_1_ fat-saturated (A and B) and axial *T*_2_ (C and D) images showing a left-sided IVC, whereby the hemiazygos vein drained into the azygos vein supradiaphragmatically, posterior to the descending aorta. Note the direct draining of the hepatic veins into the right atrium (white arrow in the last image). IVC, inferior vena cava.

**Figure 13.**
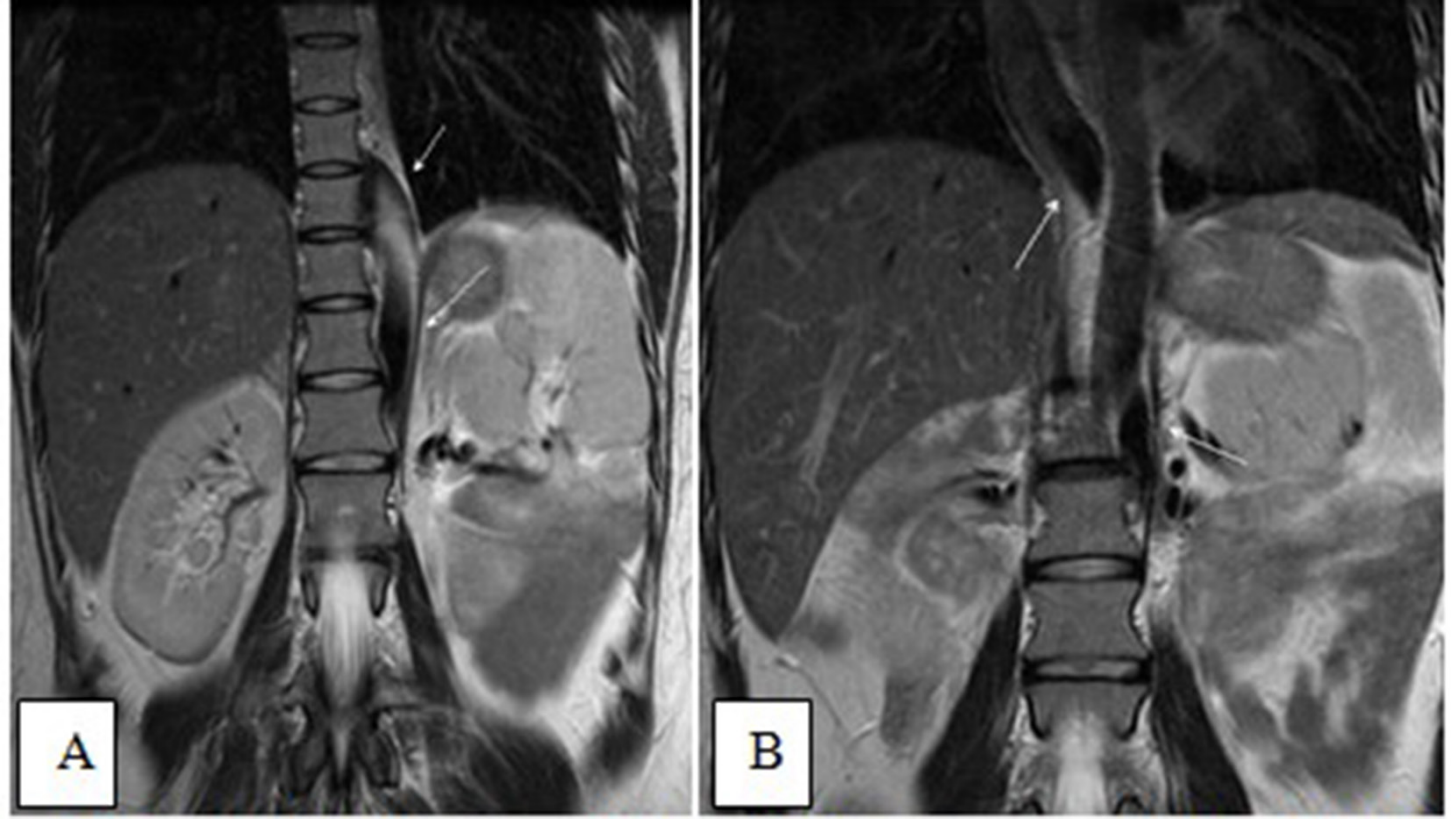
Coronal *T*_2_ weighted images (A and B) showing a left-sided IVC, continuing as a hemiazygos vein draining into the azygos vein supradiaphragmatically, posterior to the descending aorta. IVC, inferior vena cava.

In the current case, a left IVC was draining into the hemiazygos vein that eventually joined the azygos vein supradiaphragmatically; the combined hemiazygos and azygos vein was draining into the SVC (Figures 7–13). This pattern is not consistent with the common left IVC variations reported in the literature. In the literature, we were not able to find data on the prevalence of left hemiazygos/azygos variations.^2^ We believe that the left IVC with hemiazygos and azygos continuation is a very rare variation of IVC.

Haswell et al reported a left IVC with azygos and accessory hemiazygos that eventually entered the SVC through the brachiocephalic route.^14^ Koç et al reported a similar variation, but the drainage promptly returned to the continuous azygos vein and progressed thereafter.^15^

The Riedel lobe was first described by Corbin in 1830; it was subsequently defined by Riedel in 1888 as a “round tumor on the anterior side-of the liver, near the gallbladder, to its right.” It is more common in females. With the advancements made in several imaging modalities, it has become easier to detect such rare hepatic morphological variants.^16^

The Riedel lobe is either congenital or acquired. The congenital Riedel lobe results from an anomaly that occurs in the development of the hepatic bud, which leads to the formation of an accessory lobe.^17^

The acquired form of the Riedel lobe could be secondary to hepatic morphological modifications (which are either age- or sex-related) and to skeletal anomalies, such as kyphoscoliosis with a wide thorax.^18^ It might also be secondary to tractions exercised by adherential syndrome due to lithiasic cholecystitis, peritoneal inflammation, and prior surgical intervention.^19^

**Figure 14.**
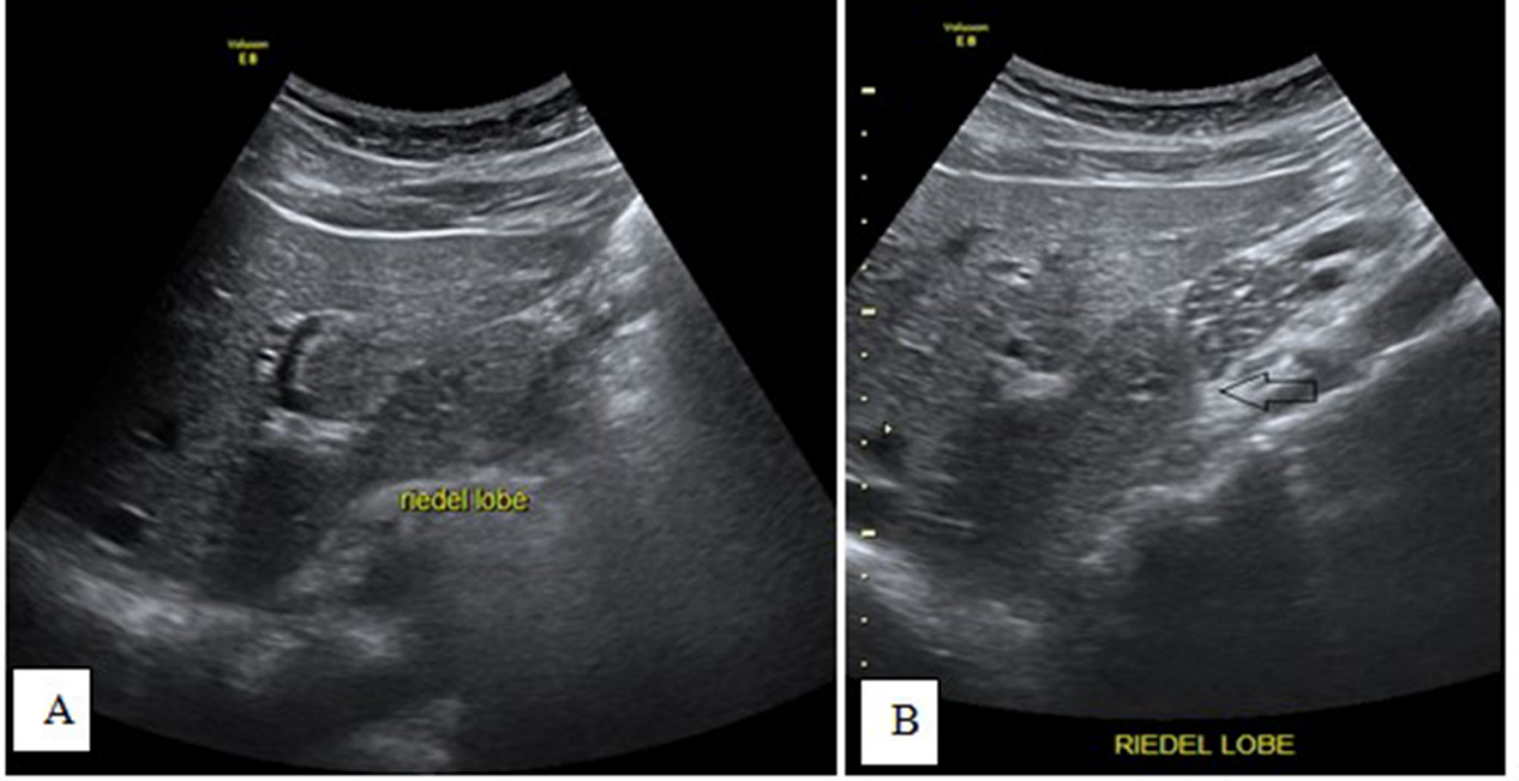
Sagittal-view ultrasound images (A and B) in the epigastric region shows the Riedel lobe as a tongue-like projection from the caudate lobe.

**Figure 15.**
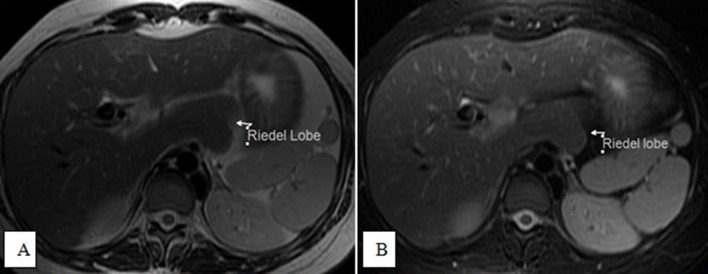
Axial *T*_2_ (A) and *T*_2_ fat-saturated (B) images showing a tongue-like projection from the caudate lobe, which shows a similar signal.

**Figure 16.**
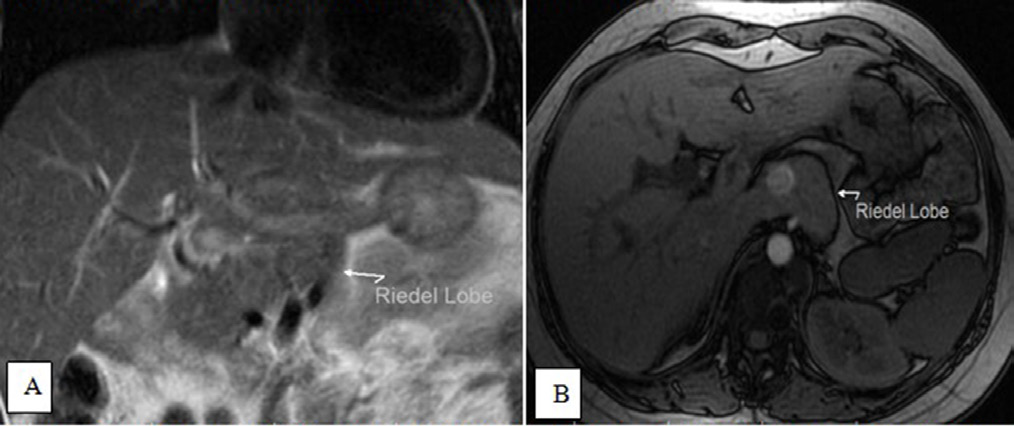
Coronal *T*_2_ (A) and axial opposed-phase weighted images (B) showing a tongue-like projection from the caudate lobe, which shows a similar signal.

Our case was typical of an accidentally discovered, non-palpable Riedel lobe of the liver, as it was discovered during an examination for a bilobed spleen (Figures 14–16). Generally, the Riedel lobe can present with minor symptoms such as abdominal discomfort due to extrinsic compression and torsion episodes, or it may be asymptomatic, as in our case. Its differential diagnosis includes all causes of a palpable normal liver. When diagnosing a Riedel lobe, all available imaging techniques can be used, such as ultrasound, CT scans, or MRI; in some cases, radionuclide imaging and arteriographic examinations can also be used.^20^

A typical Riedel lobe is usually associated with a good prognosis, particularly in cases of an early-stage diagnosis, and there is typically a lack of complications.^20^ Knowledge or suspicion of the possibility of a Riedel lobe is important, as it does not always remain clinically latent in cases of torsion or hepatic tumors, which can include metastasis or hepatocellular carcinoma that may sometimes arise only in the lowest part of the Riedel lobe.^21^

## Conclusion

We presented an extremely rare combined morphological congenital anomaly of the spleen, IVC, and liver in one case. The bilobed spleen is composed of medial (internal) and lateral (external) lobes connected at the hilum. The medial lobe is seen compressing and mildly displacing the tail of the pancreas. The left kidney is displaced downwards. The rarely encountered bilobed spleen could be confused with splenomegaly. A bilobed spleen, as reported here, might be misinterpreted as a mass originating from the tail of the pancreas, left adrenal gland, or the fundus of the stomach. The similar signal intensity to spleen on MRI is a crucial distinguishing factor.

Transposition of the IVC (also known as a left-sided IVC) drained into the hemiazygos vein, that eventually joined the azygos vein supradiaphragmatically; the combined hemiazygos and azygos veins were draining into the SVC, referring to a very rare variant course of the IVC. The findings of this report may ultimately be of importance to radiologists, surgeons, clinicians, and patients.

This type of elongation of the caudate lobe of the liver may cause symptoms like sensations of pressure, pulling, and pain in the epigastrium. Moreover, knowledge of this type of liver anomaly will be helpful for surgeons when planning hepatobiliary surgeries.

## Learning points

1. Knowledge of the possible anomalies and different morphological variants of the spleen, IVC, and liver are important in order to avoid pitfalls in the interpretation of abdominal imaging studies, such as MRI and ultrasound.
2. For this reason, this case report demonstrates an extremely rare variant of the spleen that could be confused for splenic or renal mass lesions.
3. No available appreciable data are available regarding the bilobed spleen, its related findings, or symptoms in the imaging literature.
4. This case demonstrates an extremely rare variant of the transposition of the IVC (also known as left-sided IVC) draining into the hemiazygos vein that eventually joined the azygos vein supradiaphragmatically; the combined hemiazygos and azygos veins were draining into the SVC.
5. Knowledge or suspicion of the possibility of a Riedel lobe is important, as it does not always remain clinically latent in cases of torsion or hepatic tumors, which include metastasis. Further, hepatocellular carcinoma may sometimes arise only in the lowest part of the Riedel lobe.

## References

[1] Bass JE , Redwine MD , Kramer LA , Huynh PT , Harris JH . Spectrum of congenital anomalies of the inferior vena cava: cross-sectional imaging findings . Radiograph 2000 ; 20 : 639 – 52 . doi: 10.1148/radiographics.20.3.g00ma09639 10835118

[2] Kandpal H , Sharma R , Gamangatti S , Srivastava DN , Vashisht S . Imaging the inferior vena cava: a road less traveled . Radiographics 2008 ; 28 : 669 – 89 . doi: 10.1148/rg.283075101 18480478

[3] Wolf DC . Chapter 94 Evaluation of the Size Shape, and Consistency of the Liver . : Clinical methods: the history physical, and laboratory examinations . 3rd edition . Boston : The British Institute of Radiology. ; 1990 .

[4] Couinaud C . Surgical Anatomy of the Liver Revisited . Paris : The British Institute of Radiology. ; 1989 . . 31 – 5 .

[5] Reitemeier RJ , Butt HR , Baggenstoss AH . Riedel's lobe of the liver . Gastroenterology 1958 ; 34 : 1090 – 5 . 13548342

[6] Riedel I . Ueber den zungenfrmigen Fortsatz des rechten Leberlappens und seine pathognostische Bedeutung für die Erkrankung der Gallenblase nebst Bemerkungen über Gallensteinoperationen . Berliner klinische Wochenschrift 1888 ; 25 : 577 – 602 .

[7] Zinner MJ , Schwartz SI , Ellis H . Maingot’s Abdominal Operations . : The Spleen . 10th ed . Stamford : The British Institute of Radiology. ; 1997 . . 2032 – 7 .

[8] Borley NR , Standring S . Gray’s Anatomy: The Anatomical Basis of Clinical Practice . 40th edition . Edinburg : The British Institute of Radiology. ; 2010 . . 1191 – 7 .

[9] JKT L , Sagel SS , Stanley RJ . Computed body tomography with MRI correlation . 2nd edn . New York : The British Institute of Radiology. ; 1989 . . 521 – 41 .

[10] Impellizzeri P , Montalto AS , Borruto FA , Antonuccio P , Scalfari G , Arena F , et al . Accessory spleen torsion: rare cause of acute abdomen in children and review of literature . J Pediatr Surg 2009 ; 44 : e15 – e18 . doi: 10.1016/j.jpedsurg.2009.06.029 19735802

[11] Dodds WJ , Taylor AJ , Erickson SJ , Stewart ET , Lawson TL . Radiologic imaging of splenic anomalies . AJR Am J Roentgenol 1990 ; 155 : 805 – 10 . doi: 10.2214/ajr.155.4.2119113 2119113

[12] Gayer G , Zissin R , Apter S , Atar E , Portnoy O , Itzchak Y . CT findings in congenital anomalies of the spleen . Br J Radiol 2001 ; 74 : 767 – 72 . doi: 10.1259/bjr.74.884.740767 11511506

[13] Freeman JL , Jafri SZ , Roberts JL , Mezwa DG , Shirkhoda A . CT of congenital and acquired abnormalities of the spleen . Radiographics 1993 ; 13 8316667 : 597 – 610 . doi: 10.1148/radiographics.13.3.8316667 8316667

[14] Ovalı GY , Ş Örgüç , Serter S , Göktan C , Pekindil G . Vena cava inferior anomalies on computed tomography . Türk Göğüs Kalp Damar Cer Derg 2006 ; 14 : 169 – 71 .

[15] Koc Z , Oguzkurt L . Interruption or congenital stenosis of the inferior vena cava: prevalence, imaging, and clinical findings . Eur J Radiol 2007 ; 62 : 257 – 66 . doi: 10.1016/j.ejrad.2006.11.028 17161574

[16] Sham R , Sain A , Silver L . Hypertrophic riedel’s lobe of the liver . Clin Nucl Med 1978 ; 3 : 79 – 81 . doi: 10.1097/00003072-197803000-00001 657671

[17] Aljabri B , MacDonald PS , Satin R , Stein LS , Obrand DI , Steinmetz OK , Ps M , Ls S , Di O , Ok S . Incidence of major venous and renal anomalies relevant to aortoiliac surgery as demonstrated by computed tomography . Ann Vasc Surg 2001 ; 15 : 615 – 8 . doi: 10.1007/s10016-001-0095-7 11769141

[18] Actan ZA , Savas R , Pinar Y , et al . Lobe and segment anomalies of the liver . J Anat Soc India 2001 ; 50 : 15 .

[19] Yano K , Ohtsubo M , Mizota T , Kato H , Hayashida Y , Morita S , et al . Riedel's lobe of the liver evaluated by multiple imaging modalities . Intern Med 2000 ; 39 : 136 – 8 . doi: 10.2169/internalmedicine.39.136 10732830

[20] Chaulieu C , Claudon M , Régent D , Tréheux A . Riedel's lobe. Echotomographic aspects . J Radiol 1982 ; 63 : 637 . 7153958

[21] Champetier J , Yver R , Létoublon C , Vigneau B . A general review of anomalies of hepatic morphology and their clinical implications . Anat Clin 1985 ; 7 : 285 – 99 . doi: 10.1007/BF01784645 3833290

